# Prevalence of Suboptimal Health Status and the Relationships between Suboptimal Health Status and Lifestyle Factors among Chinese Adults Using a Multi-Level Generalized Estimating Equation Model

**DOI:** 10.3390/ijerph17030763

**Published:** 2020-01-25

**Authors:** Tao Xu, Guangjin Zhu, Shaomei Han

**Affiliations:** 1 Department of Epidemiology and Statistics, Institute of Basic Medical Sciences, Chinese Academy of Medical Sciences and School of Basic Medicine, Peking Union Medical College, Beijing 100005, China; hansm1@vip.sina.com; 2 Department of Physiopathology, Institute of Basic Medical Sciences, Chinese Academy of Medical Sciences and School of Basic Medicine, Peking Union Medical College, Beijing 100005, China; zgj_1942@tom.com

**Keywords:** suboptimal health, prevalence, lifestyle, generalized estimating equation model

## Abstract

This study examined the prevalence of suboptimal health among Chinese adults based on a large-scale national survey and clarified the relationship between suboptimal health and lifestyle factors. We used multi-level generalized estimating equation models to examine the relationships between suboptimal health and lifestyle factors. Of the 48,978 respondents, 34,021 reported one or more suboptimal health symptoms, giving a suboptimal health status prevalence of 69.46%. After controlling for the cluster effect of living areas and confounding effect of demographic characteristics, factors associated with suboptimal health were: current smoking (odds ratio (OR) = 1.083, 95% confidence interval (CI): 1.055–1.111), drinking alcohol (OR = 1.075, 95% CI: 1.025–1.127), family history of disease (OR = 1.203, 95% CI: 1.055–1.111), sleeping <6 h per day (OR = 1.235, 95% CI: 1.152–1.256), poor sleep quality (OR = 1.594, 95% CI: 1.515–1.676), stress (OR = 1.588, 95% CI: 1.496–1.686), negative life events (OR = 1.114, 95% CI: 1.045–1.187), unhealthy diet choices (OR = 1.093, 95% CI: 1.033–1.156), and not regularly having meals at fixed hours (OR = 1.231, 95% CI: 1.105–1.372). Respondents who exercised regularly had lower odds of having suboptimal health status (OR = 0.913, 95% CI: 0.849–0.983). Suboptimal health has become a serious public health challenge in China. The health status of the population could be effectively improved by improving lifestyle behaviors.

## 1. Introduction

The World Health Organization defines health in its broader sense as a state of complete physical, mental, and social well-being, rather than merely as the absence of disease or infirmity [1]. With advancing understanding of health, this definition has expanded to consider suboptimal health status. Although there is currently no common definition for suboptimal health status, people usually consider suboptimal health as a borderline state between health and disease, characterized by declines in vitality, physiological function, and the capacity for adaptation, and including medically undiagnosed or functional somatic syndromes [2,3]. In China, rapid economic development has meant that many people face pressures from their work and home lives that may develop into suboptimal health status. Globally, suboptimal health has attracted increasing attention from medical professionals as an important public health issue [4,5]. It is important to properly assess suboptimal health and explore the factors associated with suboptimal health to prevent diseases and improve human health. Previous epidemiological surveys showed that 55%–75% of China’s population experienced suboptimal health [6,7,8].

Lifestyle behaviors are important factors affecting diseases and health. Many lifestyle behaviors influence a person’s ability to maintain their energy balance over the long term. For example, duration of sleep may influence energy consumption, energy expenditure, or both [9,10]. Unhealthy lifestyles are also closely related to many chronic diseases [11,12,13,14]. Chen reported that in the general population, major risk factors for suboptimal health status included poor stress management, poor self-actualization, lack of exercise, and poor interpersonal relationships [15].

Despite the increasing availability of information on this topic, more evidence is needed regarding the prevalence of suboptimal health among representative Chinese populations that cover a broad age range and include different minorities and regions. To date, most estimates of suboptimal health status have been based on a single province or small sample size, and could not represent the general Chinese adult population [6,7,8,16]. Little is known about the current prevalence of suboptimal health status in China based on large-scale representative national samples. Furthermore, people living in the same area may be likely to suffer from the effects of similar living circumstances, which may influence the accuracy and generalizability of data on the relationship between suboptimal health and lifestyle. However, current studies focused on suboptimal health have not considered and adjusted for the cluster effect of living area, which has a confusing influence on the results of studies investigating suboptimal health. Specifically, this study aimed to examine the prevalence of suboptimal health among Chinese adults. We covered a broad age range using data from a large-scale cross-sectional national survey, and clarified the relationships between suboptimal health status and lifestyle factors using multi-level generalized estimating equation (GEE) models controlled for the cluster effect of living area.

## 2. Material and Methods

### 2.1. Sample and Participants

This survey was conducted in six provinces or autonomous regions of China from 2007 to 2011: Heilongjiang province, Hunan province, Yunnan province, Inner Mongolia autonomous region, Sichuan province, and Ningxia Hui autonomous region. China is a vast multi-ethnic country with huge regional differences, populated by Han and 55 ethnic minorities. These six provinces or autonomous regions were selected using convenience sampling to cover a variety of municipal regions and include sufficient minority respondents. In each selected province or autonomous region, a two-stage cluster sampling method was used to recruit eligible respondents. Two or three cities were selected based on their population and economic conditions using a simple random sampling method. Next, dozens of communities were selected within each city using a cluster random sampling method based on population density. All residents of the selected communities were considered eligible for the survey if they were aged ≥18 years, were not suffering from serious chronic diseases, and had not run a high fever in the past 15 days. After signing informed consent forms, all respondents voluntarily attended temporary physical examination centers to take part in the survey. The survey was approved by the Review Board of the Institute of Basic Medical Sciences, Chinese Academy of Medical Sciences (No. 005-2008).

### 2.2. Suboptimal Health Assessment

The self-rating suboptimal health scale developed using a Delphi method by researchers from Medical College of Jinan University was used to assess the suboptimal health status of the community populations [17]. Previous reports showed that this scale had good reliability and repeatability [17,18,19]. The scale includes 18 symptom items grouped in six dimensions: physical symptoms, psychological symptoms, vigor, social adaptability, immunity, and going to hospital. The physical symptoms dimension contains five suboptimal health symptom items: fatigue, headache or dizziness, tinnitus, numbness or stiffness in the shoulders or legs, and a sense of pharyngeal foreign bodies. The psychological symptoms dimension contains six suboptimal health symptom items: upset, loneliness, inattention, anxiety, dreaminess, and forgetfulness. The vigor dimension contains three suboptimal health symptom items: decreased vitality, disinterest in surroundings and moodiness. The social adaptability dimension contains two suboptimal health symptom items: feeling tired at work and incompatibility with coworkers. The immunity dimension contains one suboptimal health symptom item: susceptibility to flu or other diseases. The going to hospital dimension contains one suboptimal health symptom item: the feeling of suffering from undiagnosed diseases. Respondents that had experienced one or more symptom item for more than one month in the past year were considered as having suboptimal health status.

### 2.3. Definition of Covariates

Covariates included demographic characteristics (e.g., age, gender, occupation, marital status, educational level) and lifestyle factors such as smoking, alcohol drinking, ethnicity, family history of cardiovascular or cerebrovascular diseases, body mass index (BMI), sleep duration, sleep quality, stress, life events, hypertension, exercise, diet choices, and meal times.

Respondents were categorized into three age groups: youth (18–34 years), middle aged (35–59 years), and older adults (≥60 years old). Occupation was classified as blue-collar worker and “white-collar worker” (e.g., teacher, doctor, other professionals, students, governmental and institute employees). Marital status was categorized as married, single, divorced, or widowed. Educational level was categorized into three groups: primary school, middle school, and college. Ethnicity included Han, Yi, Miao, Mongolia, Tibetan, Korean, Hui, Tujia, and others. BMI was defined as weight in kg divided by height in m^2^. Weight was measured to the nearest 0.1 kg and height was measured to the nearest 1 mm. According to Chinese guidelines for the prevention and control of adult overweight and obesity and the Working Group on Obesity in China criteria [20], normal weight was defined as a BMI <24 kg/m^2^, overweight as a BMI of 24–27.9 kg/m^2^, and obesity as a BMI ≥28 kg/m^2^.

Sleep duration was dichotomized into >6 h of sleep and <6 h of sleep. Respondents were also asked to self-report whether they had good sleep quality. Stress was defined as self-perceived economic, life, or work stress. Life events were defined by whether the respondent had recently experienced negative or positive events. Negative events included loss of job, retirement, loss of crops/business failure, burglary, marital separation/divorce, other major intra-family conflict, major personal injury or illness, violence, death of a spouse, death/major illness of another close family member, and other major stressors. Positive events included events such as the wedding of a family member, new job, or a birth in the family. Diet choice was defined as whether the respondent had unhealthy diet choice preferences, such as food that was highly spicy, sweet, salty, or greasy. Meal time was used to indicate whether the respondent regularly had breakfast, lunch, and dinner at fixed times.

Blood pressure was measured in the morning after respondents had rested for 5 min in a seated position with their back supported, feet on the floor, and right arm supported with the cubit fossa at heart level. The appropriate cuff was chosen based on the respondent’s arm circumference. OMRON HEM-7000 electronic sphygmomanometers (OMRON Health-Care, Kyoto, Japan) were used to measure blood pressure. Hypertension was defined as not being on antihypertensive medications and having systolic blood pressure of ≥140 mmHg or diastolic blood pressure of ≥90 mmHg [21,22].

### 2.4. Quality Control

This study adhered to strict quality control standards. All researchers were trained based on a training manual. A preliminary survey was conducted to confirm that the investigators were conducting the survey correctly. Trained medical professionals conducted the survey and respondent interviews. All case report forms were double-checked to guarantee the authenticity and accuracy of raw data. The survey database was constructed with EPI3.02 software (EpiData Association, Odense, Denmark), with data input checked twice by two data managers to guarantee the accuracy and integration of the data.

### 2.5. Statistical Analysis

Statistical analysis was performed with SAS9.4 software (SAS institute Inc., Cary, NC, USA). A two-tailed *p*-value < 0.05 was defined as statistically significant. Continuous data were described using mean and standard deviation when normally distributed, and median (lower quartile, upper quartile) when the distribution was skewed. Categorical data were described with number and percentage and compared using chi-square tests. Considering that respondents living in the same city were likely to suffer the effects of similar living circumstances, multi-level GEE models were used to examine the relationships between suboptimal health status and lifestyle factors to control for the cluster effect of living area and the confounding effect of demographic characteristics. Odds ratios and 95% confidence intervals were used to assess the strength of these relationships.

## 3. Results

In total, 52,265 respondents signed an informed consent form and were willing to participate in this survey. Of these, 48,978 respondents completed all survey scales, giving a completion rate of 93.7%.

The average age of all respondents was 44.3 ± 16.8 years, and 59.5% respondents were female. In total, 46.7% of respondents were white-collar workers, such as teachers, doctors, other professionals, students, and governmental and institute employees. Widowed or divorced respondents accounted for 5.45% of the sample. The majority of respondents (74.9%) were of Han nationality. The percentages of regular smokers and drinkers were 22.5% and 22.3%, respectively. The percentages of overweight and obese respondents were 31.2% and 12.0%, respectively. In addition, 782 respondents reported feeling high levels of work, home, or economic stress, and 2058 respondents had recently experienced positive or negative life events. Just under half (45.87%) of the respondents exercised regularly, 13.30% slept for <6 h per day, and only 28.33% reported good sleep quality. The majority of respondents (67.67%) had special dietary preferences and 9.84% did not regularly have meals at fixed hours.

Of the 48,978 respondents, 34,021 reported one or more suboptimal health symptoms, giving a suboptimal health status prevalence rate of 69.46%. The median number of suboptimal health symptoms was 2 (0, 5). The most common suboptimal health symptoms were forgetfulness (38.53%), dreaminess (30.89%), and headache or dizziness (30.10%). Table 1 shows the prevalence of each the 18 suboptimal health symptoms for the whole sample, and by sex and age groups.

The average age of respondents with a suboptimal health status was 45.0 ± 16.7 years and that of respondents without suboptimal health was 42.6 ± 17.0 years. The prevalence rate of suboptimal health status was 65.02% for youths, 71.08% for middle-aged adults, and 72.48% for older adults. The prevalence rate of suboptimal health status for males (67.74%) was lower than that for females (72.67%) (*p* < 0.0001). The prevalence rate of suboptimal health status was statistically significantly different among various ethnicities; respondents from Tujia had the lowest prevalence of suboptimal health status (60.21%) and those from Yi had the highest prevalence (87.15%). Blue-collar workers had a higher prevalence (73.71%) of suboptimal health status than their white-collar worker counterparts. The prevalence rate of suboptimal health status declined as educational level increased (*p* < 0.0001). The prevalence rate of suboptimal health status for widowed or divorced respondents (77.11%) was significantly higher than that among married or single respondents.

Compared with respondents without a family history of cardiovascular or cerebrovascular diseases, respondents with a family history of disease had a higher prevalence rate of suboptimal health status (79.51% vs. 67.89%). Compared with respondents with normal blood pressure, hypertensive respondents had a higher prevalence rate of suboptimal health status (71.83% vs. 68.45%). We observed higher prevalence rates of suboptimal health status among respondents with short sleep duration (82.73% vs. 67.42%) and poor sleep quality (72.64% vs. 61.41%) compared with their counterparts. Respondents who reported experiencing stress had a higher prevalence rate of suboptimal health status than their counterparts without stress (86.96% vs. 69.18%), as did those who had experienced negative life events (79.71% vs. 69.09%) or positive life events (78.02% vs. 69.38%). Respondents who had unhealthy diet choices had a higher prevalence rate of suboptimal health status than their counterparts (70.73% vs. 66.80%). Respondents who exercised regularly had a lower prevalence rate of suboptimal health status than those who did not exercise regularly (65.14% vs. 73.13%), as did those who had regular meals at fixed hours (68.21% vs. 80.94%). There were no significant differences between different smoking conditions, drinking conditions, and BMI groups. The prevalence rates of suboptimal health status by different demographic characteristics and lifestyle factors are detailed in Table 2.

Table 3 presents the results of the bivariate and multivariate multi-level GEE models factors associated with suboptimal health status. After controlling for the cluster effect of living area, we found that gender, occupation, marital status, educational level, smoking, alcohol drinking, ethnicity, family history of cardiovascular or cerebrovascular diseases, sleep duration, sleep quality, stress, negative life events, exercise, diet choice, and meal times were associated with suboptimal health status. Table 4 and Table 5 show the impact of independent variables on each dimension of suboptimal health separately based on multi-level GEE models. Independent associated factors were similar for six dimensions of suboptimal health, as shown in Table 4 and Table 5.

## 4. Discussion

In multi-stage sampling, survey data have a hierarchical structure in which individuals are nested within higher level sampling units. Hierarchical social structures naturally generate multi-level data in which the lower level units are nested in the next higher level units. Individual suboptimal health status is determined by individual characteristics (e.g., age, ethnicity, education, and occupation at the micro level) as well as features of the social contexts or social environments in which individuals live (e.g., culture or subculture). Factors such as diet choice/preferences at the macro level can also produce measurable effects. People living in the same neighborhood may share similarities because they are influenced by the same neighborhood socioeconomic characteristics. In statistical terms, there is within-group homogeneity and between-group heterogeneity in hierarchically structured data [23]. Traditional analytical methods such as logistic regression assume that observations are independently but identically distributed. Violation of this assumption results in incorrect inferences in statistical analyses. Multi-level GEE models provide an appropriate analytical framework to deal with observation dependence in multi-level data. More importantly, multi-level models permit exploration of the nature and extent of relationships at both micro and macro levels, as well as across levels [23]. We considered that individuals living in the same area were likely to suffer from the effects of similar living circumstances, which would have an inevitable confusing influence on the accuracy and generalization of the study results concerning suboptimal health status. This was the first study to explore the prevalence of suboptimal health status and the relationships of suboptimal health status and lifestyle factors using multi-level GEE models controlled for the cluster effect of the same living area and similar living circumstances to guarantee the accuracy and generalizability of the study results.

We found that more than two-thirds of Chinese adults had suboptimal health status. The prevalence of suboptimal health status in this study was similar to that in several previous reports [6,7,24], but significantly higher than that in a report by Wang et al. [25,26]. Wang et al. reported that the prevalence of suboptimal health status was 9.0%, based on the Suboptimal Health Status Questionnaire-25 (SHSQ-25) administered in a cross-sectional survey with 4313 participants [25]. The low prevalence reported by Wang et al. was mainly attributable to different participant selection criteria and a higher threshold for scores. Suboptimal health is often assessed with an assessment scale, such as the Delphi suboptimal health scale used in our study and the SHSQ-25 [25,26]. The SHSQ-25 contains 25 items under five domains (fatigue, the cardiovascular system, the digestive tract, the immune system, and mental status). Participants are asked to rate specific statements on a five-point scale, with raw scores of 1–5 recorded as 0–4. Total scores are calculated for each participant by summing the ratings for the 25 items. The 25 SHSQ-25 items are similar as to the 18 symptoms covered in Delphi scale, although the SHSQ-25 items are more delicate. With the SHSQ-25, a high score (≥35) represents poor health, and is regarded as suboptimal health status [25,26]. In other words, having more than one-third of the total possible score (100) in the SHSQ-25 was considered to reflect suboptimal health status. In our study, respondents were considered as having suboptimal health status if they suffered from more than one-third of the 18 symptoms (≥7 symptoms); the prevalence of suboptimal health status was 19.24%. In addition, Wang et al. [25] included 4313 participants aged 18–65 years, whereas participants currently suffering from diabetes, hypertension, hyperlipidemia, cardiovascular or cerebrovascular conditions, any type of cancer, and gout were excluded. In our study, 13.07% of respondents were aged over 65 years and we only excluded those who suffered from serious chronic diseases and had run a high fever in the past 15 days. Older participants and those with mild chronic diseases were also more likely to be classified as having suboptimal health status than other participants, which may explain the higher prevalence found in our study. If those who were aged over 65 years or currently suffering from mild chronic diseases were excluded, the prevalence of suboptimal health status might be similar to that in the previous report.

Unhealthy lifestyles were closely related to both chronic diseases and suboptimal health status. In a recent report, Ma et al. noted that good sleep quality, abundant physical exercise, and adequate nutrition intake were negatively associated with suboptimal health status, whereas overuse of electronic devices and weight loss were positively associated with suboptimal health status [24]. Chen et al. reported that poor stress management, poor self-actualization, lack of exercise, and poor interpersonal relationships were positively associated with suboptimal health status [15]. Suboptimal health has been observed to be related to low levels of physical activity and exercise, which are known risk factors for cardiovascular and cerebrovascular diseases [27]. Smoking and drinking alcohol have been confirmed as associated with many chronic diseases [28,29,30,31] and were also associated with suboptimal health status in our study. Sleep was defined as a natural and reversible state of reduced responsiveness to external stimuli and relative inactivity, accompanied by a loss of consciousness. Sleep problems and sleep continuity were significant predictors of relapse. Sleep deprivation and sleep disruptions may cause severe cognitive and emotional problems [32,33]. Previous studies reported that inadequate sleep duration caused heightened cardiovascular risk, cerebrovascular disease, psychiatric disorders, and obesity [34,35,36,37].

In addition to common unhealthy lifestyle factors, such as lack of exercise, smoking, drinking alcohol, short sleep duration, and poor sleep quality, we found that stress, negative life events, family history of cardiovascular or cerebrovascular diseases, unhealthy diet choices, and irregular meal times were associated with a higher prevalence risk of suboptimal health.

Wu et al. reported that poor work-recreation balance was associated with increased risk for suboptimal health status [38]. Chang et al. found that suboptimal health status among middle school teachers was closely related to occupational stress [39]. Negative life events and work, home, or economic stress were important factors for depression and anxiety, and may further lead to suboptimal health status. Respondents with parents that suffered from cardiovascular or cerebrovascular diseases may experience anxiety about suffering these diseases themselves, which may in turn make them more susceptible to suboptimal health status.

We found that both unhealthy diet choices and irregular meal times were closely related to a higher prevalence of suboptimal health. Owen et al. confirmed the role of diet and nutrition in mental health and wellbeing [40]. In addition, a high-salt diet was reported to be closely associated with hypertension [41]. Cordner et al. reported that the associations between consumption of a high-fat or a “Western” diet and metabolic disorders (e.g., obesity, diabetes, and cardiovascular disease) have long been recognized, and a large body of evidence suggests that diets high in fat could also have a profound impact on the brain, behavior, and cognition [42]. A large prospective study found the habitual consumption of spicy foods was inversely associated with total and certain cause specific mortality, independent of other risk factors for death [43]. Regularly having breakfast, lunch, and dinner at fixed times may induce individuals to work and live within a routine, which may help them to maintain healthy living habits and timetables, which could benefit their physical and psychological health and in turn help to prevent anxiety and suboptimal health status.

This study had several limitations that should be mentioned. First, because of the cross-sectional design, it was not possible to confirm causal relationships between suboptimal health and lifestyle factors. Second, suboptimal health has several different definitions, which limited the comparison of our study results with other reports on the prevalence of suboptimal health. Third, lifestyle factors were self-reported in this study, which reduced the precision of measurement of these factors, such as smoking, alcohol drinking, sleep habits, diet choices, and meal times. Finally, since the survey data referred to years 2007–2011 and the study findings reflected the situation in 2007–2011, they may not reflect the current situation.

## 5. Conclusions

Despite these limitations, our findings showed that suboptimal health has become a serious public health challenge in China. Unhealthy lifestyles were closely associated with suboptimal health, with key factors being smoking, drinking alcohol, short sleep duration, poor sleep quality, lack of regular exercise, stress, negative life events, family history of cardiovascular and cerebrovascular diseases, unhealthy diet choices, and irregular meal times. The health status of the population could be effectively improved by improving lifestyle behaviors.

## Figures and Tables

**Table 1:** Prevalence (%) of 18 suboptimal health symptoms by sex and age groups.

Items	Total (*n* = 48,978)	Sex		Age Groups		
		Men (*n* = 19,834)	Women (*n* = 29,144)	18–34 y (*n* = 15,430)	35–59 y (*n* = 23,372)	≥60 y (*n* = 10,176)
Fatigue	14,300 (29.20%)	5142 (25.93%)	9158 (31.42%)	4261 (27.62%)	7264 (31.08%)	2775 (27.27%)
Headache or dizziness	14,740 (30.10%)	4474 (22.56%)	10,266 (35.23%)	3135 (20.32%)	8068 (34.52%)	3537 (34.76%)
Tinnitus	8415 (17.18%)	3169 (15.98%)	5246 (18.00%)	1248 (8.09%)	4621 (19.77%)	2546 (25.02%)
Numbness or stiffness in the shoulders or legs	10,324 (21.08%)	3440 (17.34%)	6884 (23.62%)	1498 (9.71%)	5861 (25.08%)	2965 (29.14%)
A sense of pharyngeal foreign bodies	8205 (16.75%)	3324 (16.76%)	4881 (16.75%)	2738 (17.74%)	4037 (17.27%)	1430 (14.05%)
Upset	10,192 (20.81%)	3050 (15.38%)	7142 (24.51%)	3485 (22.59%)	4987 (21.34%)	1720 (16.90%)
Loneliness	4238 (8.65%)	1547 (7.80%)	2691 (9.23%)	1890 (12.25%)	1573 (6.73%)	775 (7.62%)
Inattention	9944 (20.30%)	3515 (17.72%)	6429 (22.06%)	3897 (25.26%)	4206 (18.00%)	1841 (18.09%)
Anxiety	7064 (14.42%)	2323 (11.71%)	4741 (16.27%)	2513 (16.29%)	3272 (14.00%)	1279 (12.57%)
Dreaminess	15,131 (30.89%)	4733 (23.86%)	10,398 (35.68%)	4478 (29.02%)	7544 (32.28%)	3109 (30.55%)
Forgetfulness	18,872 (38.53%)	6601 (33.28%)	12,271 (42.10%)	4579 (29.68%)	9641 (41.25%)	4652 (45.72%)
Decreased vitality	9872 (20.16%)	3645 (18.38%)	6227 (21.37%)	2366 (15.33%)	4643 (19.87%)	2863 (28.13%)
Disinterest in surroundings	6514 (13.30%)	2361 (11.90%)	4153 (14.25%)	2248 (14.57%)	2928 (12.53%)	1338 (13.15%)
Moodiness	7179 (14.66%)	2388 (12.04%)	47,911 (16.44%)	2761 (17.89%)	3215 (13.76%)	1203 (11.82%)
Feeling tired at work	5279 (10.78%)	2048 (10.33%)	3231 (11.09%)	1753 (11.36%)	2627 (11.24%)	899 (8.83%)
Incompatibility with coworkers	1588 (3.24%)	630 (3.18%)	958 (3.29%)	523 (3.39%)	765 (3.27%)	300 (2.95%)
Susceptibility to flu or other diseases	8463 (17.28%)	2849 (14.36%)	5614 (19.26%)	2333 (15.12%)	4104 (17.56%)	2026 (19.91%)
The feeling of suffering from undiagnosed diseases	7253 (14.81%)	2580 (13.01%)	4673 (16.03%)	2009 (13.02%)	3743 (16.01%)	1501 (14.75%)

**Table 2:** Prevalence (%) of suboptimal health by respondents’ demographic characteristics.

Characteristics	Total	Sub-Health		Chi-Square Test	
		Yes	No	Chi-Square	*p* *
All Subjects	48,978	34,021 (59.46%)	14,957 (30.54%)		
Age (years)				215.8919	<0.0001
18–34	15,430	10,033 (65.02%)	5397 (34.98%)		
35–59	23,372	16,612 (71.08%)	6760 (28.92%)		
≥60	10,176	7376 (72.48%)	2800 (27.52%)		
Gender				349.9880	<0.0001
Male	19,834	12,841 (64.74%)	6993 (35.26%)		
Female	29,144	21,180 (72.67%)	7964 (27.33%)		
Occupation				474.5250	<0.0001
Blue-collar worker	26,101	19,238 (73.71%)	6863 (26.29%)		
White-collar worker	22,877	14,783 (64.62%)	8094 (35.38%)		
Marriage status				288.7890	<0.0001
Married	36,337	25,672 (70.65%)	10,665 (29.35%)		
Single	9972	6291 (63.09%)	3681 (36.91%)		
Widowed or divorced	2669	2058 (77.11%)	611 (22.89%)		
Education level				404.9562	<0.0001
Primary school	10,881	7709 (76.47%)	2372 (23.53%)		
Middle school	22,597	15,758 (69.73%)	6839 (30.27%)		
College	16,300	10,554 (64.75%)	5746 (35.25%)		
Smoker				1.8027	0.1794
No	37,966	26,429 (69.61%)	11,537 (30.39%)		
Yes	11,012	7592 (68.94%)	3420 (31.06%)		
Alcohol drinker				2.5979	0.1070
No	38,065	26,509 (69.64%)	11,556 (30.36%)		
Yes	10,913	7512 (68.84%)	3401 (31.16%)		
Ethnicity				436.9001	<0.0001
Han	36,663	25,242 (68.85%)	11,421 (31.15%)		
Yi	2451	2136 (87.15%)	315 (12.85%)		
Miao	675	424 (62.81%)	251 (37.19%)		
Mongolia	1802	1260 (69.92%)	542 (30.08%)		
Tibetan	896	600 (66.96%)	296 (33.04%)		
Korean	1574	1089 (69.19%)	485 (30.81%)		
Hui	2722	1865 (68.52%)	857 (31.48%)		
Tujia	1259	758 (60.21%)	501 (39.79%)		
Others	936	647 (69.12%)	289 (30.88%)		
Family history				364.8801	<0.0001
No	42,354	28,754 (67.89%)	13,600 (32.11%)		
Yes	6624	5267 (79.51%)	1357 (20.49%)		
BMI				5.7840	0.0555
Normal	27,817	19,224 (69.11%)	8593 (30.89%)		
Overweight	15,284	10,645 (69.65%)	4639 (30.35%)		
Obesity	5877	4152 (70.65%)	1725 (29.35%)		
Sleep duration				624.2102	<0.0001
≥6 h	42,462	28,630 (67.42%)	13,832 (32.58%)		
<6 h	6516	5391 (82.73%)	1125 (17.27%)		
Sleep quality				591.6443	<0.0001
Good	13,874	8520 (61.41%)	5354 (38.59%)		
Poor	35,104	25,501 (72.64%)	9603 (27.36%)		
Stress				114.6625	<0.0001
No	48,196	33,341 (69.18%)	14,855 (30.85%)		
Yes	782	680 (86.96%)	102 (13.04%)		
Negative life event				89.0887	<0.0001
No	47,243	32,638 (69.09%)	14,605 (30.91%)		
Yes	1735	1383 (79.71%)	352 (20.29%)		
Positive life event				15.8650	<0.0001
No	48,523	33,666 (69.38%)	14,857 (30.62%)		
Yes	455	355 (78.02%)	100 (21.98%)		
Hypertension				55.3106	<0.0001
No	34,287	23,469 (68.45%)	10,818 (31.55%)		
Yes	14,691	10,552 (71.83%)	4139 (28.17%)		
Regular exercise				365.9445	<0.0001
No	26,513	19,388 (73.13%)	7125 (26.87%)		
Yes	22,465	14,633 (65.14%)	7832 (34.86%)		
Diet choice				78.194	<0.0001
Routine	15,834	10,577 (66.80%)	5257 (33.20%)		
Unhealthy	33,144	23,444 (70.73%)	9700 (29.27%)		
Meal time				331.9804	<0.0001
Regular	44,157	30,119 (68.21%)	14,038 (31.79%)		
Irregular	4821	3902 (80.94%)	919 (19.06%)		

* Family history: family history of cardiovascular or cerebrovascular diseases.

**Table 3:** Risk factors associated with suboptimal health based on a multi-level GEE model.

Characteristics	Bivariate		Multivariate	
	OR	95% CI	OR	95% CI
Age (years)				
18–34	1.000	-	1.000	-
35–59	1.322	1.266–1.381	1.040	0.977–1.108
≥60	1.417	1.342–1.497	1.067	0.979–1.163
Gender				
Male	1.000	-	1.000	-
Female	1.448	1.393–1.506	1.284	1.198–1.377
Occupation				
Blue-collar worker	1.535	1.477–1.595	1.059	1.011–1.109
White-collar worker	1.000	-	1.000	-
Marriage status				
Married	1.000	-	1.000	-
Single	0.710	0.678–0.744	1.021	0.936–1.113
Widowed or divorced	1.399	1.276–1.537	1.106	1.064–1.149
Education level				
Primary school	1.000	-	1.000	-
Middle school	0.709	0.672–0.748	0.856	0.812–0.903
College	0.565	0.534–0.598	0.791	0.730–0.856
Smoker				
No	1.000	-	1.000	-
Yes	0.969	0.926–1.015	1.083	1.055–1.111
Alcohol drinker				
No	1.000	-	1.000	-
Yes	0.963	0.920–1.008	1.075	1.025–1.127
Ethnicity				
Han	1.000	-	1.000	-
Yi	3.068	2.725–3.466	1.002	0.921–1.089
Miao	0.764	0.653–0.896	0.927	0.874–0.983
Mongolia	1.052	0.949–1.167	0.824	0.801–0.847
Tibetan	0.917	0.797–1.057	0.852	0.816–0.890
Korean	1.016	0.911–1.134	0.981	0.929–1.036
Hui	0.985	0.906–1.071	1.054	1.003–1.108
Tujia	0.685	0.610–0.768	1.029	0.950–1.114
Others	1.013	0.881–1.167	0.966	0.915–1.020
Family history				
No	1.000	-	1.000	-
Yes	1.836	1.724–1.956	1.203	1.152–1.256
BMI				
Normal	1.000	-	1.000	-
Overweight	1.026	0.983–1.071	0.981	0.948–1.014
Obesity	1.076	1.012–1.144	0.998	0.965–1.032
Sleep duration				
≥6 h	1.000	-	1.000	-
<6 h	2.315	2.165–2.478	1.235	1.183–1.290
Sleep quality				
Good	1.000	-	1.000	-
Poor	1.669	1.601–1.739	1.594	1.516–1.677
Stress				
No	1.000	-	1.000	-
Yes	2.970	2.422–3.681	1.588	1.496–1.686
Negative life event				
No	1.000	-	1.000	-
Yes	1.758	1.563–1.982	1.144	1.045–1.187
Positive life event				
No	1.000	-	1.000	-
Yes	1.566	1.259–1.967	1.014	0.973–1.056
Hypertension				
No	1.000	-	1.000	-
Yes	1.175	1.126–1.226	1.000	0.977–1.022
Regular exercise				
No	1.000	-	1.000	-
Yes	0.687	0.661–0.714	0.913	0.849–0.983
Diet choice				
Routine	1.000	-	1.000	-
Unhealthy	1.201	1.153–1.251	1.093	1.033–1.156
Meal time				
Regular	1.000	-	1.000	-
Irregular	1.979	1.838–2.133	1.231	1.105–1.372

OR: odds ratio; CI: confidence interval; GEE: generalized estimating equation; Family history: family history of cardiovascular or cerebrovascular diseases.

**Table 4:** Risk factors associated with each dimension of suboptimal health based on a multi-level multivariate GEE model.

Characteristics	Physical Symptom		Psychological Symptom			Vigor	
	OR	95% CI	OR	95% CI	OR		95% CI
Age (years)							
18–34	1.000	-	1.000	-	1.000		-
35–59	1.119	1.055–1.185	1.000	0.938–1.065	1.004		0.917–1.099
≥60	1.143	1.055–1.238	0.996	0.912–1.088	1.170		1.023–1.339
Gender							
Male	1.000	-	1.000	-	1.000		-
Female	1.260	1.175–1.353	1.336	1.243–1.436	1.246		1.166–1.331
Occupation							
Blue-collar worker	1.086	1.040–1.133	1.047	0.998–1.098	1.032		0.956–1.114
White-collar worker	1.000	-	1.000	-	1.000		-
Marriage status							
Married	1.000	-	1.000	-	1.000		-
ingle	0.790	0.696–0.897	1.156	1.065–1.256	1.167		1.045–1.304
Widowed/divorced	1.056	1.021–1.093	1.162	1.107–1.220	1.165		1.101–1.232
Education level							
Primary school	1.000	-	1.000	-	1.000		-
Middle school	0.836	0.789–0.887	0.913	0.866–0.962	0.870		0.803–0.941
College	0.746	0.681–0.818	0.855	0.790–0.924	0.855		0.778–0.940
Smoker							
No	1.000	-	1.000	-	1.000		-
Yes	1.125	1.086–1.165	1.038	1.011–1.066	1.072		1.024–1.121
Alcohol drinker							
No	1.000	-	1.000	-	1.000		-
Yes	1.044	1.006–1.083	1.095	1.040–1.153	1.111		1.027–1.202
Ethnicity							
Han	1.000	-	1.000	-	1.000		-
Yi	1.003	0.873–1.152	1.039	0.949–1.138	0.901		0.832–0.976
Miao	0.922	0.878–0.968	0.946	0.874–1.025	0.880		0.814–0.952
Mongolia	0.904	0.881–0.927	0.781	0.744–0.820	0.703		0.626–0.790
Tibetan	0.944	0.891–1.000	0.722	0.683–0.763	0.731		0.665–0.804
Korean	0.986	0.927–1.050	0.915	0.876–0.957	0.950		0.829–1.089
Hui	1.066	0.999–1.137	1.046	0.997–1.097	1.046		0.983–1.112
Tujia	1.015	0.978–1.054	1.039	0.942–1.147	1.010		0.891–1.145
Others	0.910	0.820–1.011	0.986	0.942–1.147	1.014		0.971–1.059
Disease family history							
No	1.000	-	1.000	-	1.000		-
Yes	1.196	1.141–1.255	1.196	1.152–1.242	1.202		1.142–1.265
BMI							
Normal	1.000	-	1.000	-	1.000		-
Overweight	1.001	0.965–1.038	0.976	0.942–1.011	0.973		0.928–1.021
Obesity	1.041	1.003–1.080	0.985	0.950–1.020	0.969		0.917–1.025
Sleep duration							
≥6 h	1.000	-	1.000	-	1.000		-
<6 h	1.232	1.180–1.286	1.268	1.222–1.315	1.204		1.150–1.260
Sleep quality							
Good	1.000	-	1.000	-	1.000		-
Poor	1.398	1.332–1.468	1.814	1.696–1.941	1.556		1.473–1.643
Stress							
No	1.000	-	1.000	-	1.000		-
Yes	1.400	1.327–1.477	1.558	1.411–1.721	2.033		1.830–2.258
Negative life event							
No	1.000	-	1.000	-	1.000		-
Yes	1.048	0.912–1.206	1.130	1.102–1.158	1.180		1.091–1.276
Positive life event							
No	1.000	-	1.000	-	1.000		-
Yes	0.904	0.826–0.989	1.069	1.024–1.114	1.109		1.024–1.202
Hypertension							
No	1.000	-	1.000	-	1.000		-
Yes	1.014	0.994–1.034	0.986	0.966–1.006	1.014		0.971–1.059
Regular exercise							
No	1.000	-	1.000	-	1.000		-
Yes	0.945	0.879–1.015	0.925	0.867–0.987	0.824		0.746–0.910
Diet choice							
Routine	1.000	-	1.000	-	1.000		-
Unhealthy	1.062	1.001–1.127	1.107	1.050–1.167	1.145		1.064–1.232
Meal time							
Regular	1.000	-	1.000	-	1.000		-
Irregular	1.130	0.896–1.426	1.256	1.193–1.322	1.317		1.262–1.375

OR: odds ratio; CI: confidence interval; GEE: generalized estimating equation; Family history: family history of cardiovascular or cerebrovascular diseases. The physical symptoms dimension contains five items: fatigue, headache or dizziness, tinnitus, numbness or stiffness in the shoulders or legs, and a sense of pharyngeal foreign bodies. The psychological symptoms dimension contains six items: upset, loneliness, inattention, anxiety, dreaminess, and forgetfulness. The vigor dimension contains three items: decreased vitality, disinterest in surroundings, and moodiness.

**Table 5:** Risk factors associated with each dimension of suboptimal health based on a multi-level multivariate GEE model.

Characteristics	Social Adaptability		Immunity		Immunity andGoing to Hospital	
	OR	95% CI	OR	95% CI	OR	95% CI
Age (years)						
18–34	1.000	-	1.000	-	1.000	-
35–59	0.950	0.863–1.046	0.978	0.902–1.060	1.076	0.969–1.194
≥60	0.757	0.635–0.903	1.120	1.022–1.227	1.031	0.896–1.186
Gender						
Male	1.000	-	1.000	-	1.000	-
Female	1.099	1.003–1.205	1.306	1.216–1.403	1.276	1.103–1.477
Occupation						
Blue-collar worker	0.943	0.842–1.055	1.146	1.081–1.215	1.119	1.038–1.206
White-collar worker	1.000	-	1.000	-	1.000	-
Marriage status						
Married	1.000	-	1.000	-	1.000	-
Single	1.125	0.972–1.303	0.939	0.786–1.123	0.947	0.826–1.087
Widowed/divorced	1.115	1.035–1.202	0.989	0.911–1.074	0.995	0.894–1.107
Education level						
Primary school	1.000	-	1.000	-	1.000	-
Middle school	0.774	0.706–0.849	0.740	0.661–0.828	0.756	0.660–0.866
College	0.622	0.553–0.700	0.655	0.544–0.790	0.764	0.673–0.867
Smoker						
No	1.000	-	1.000	-	1.000	-
Yes	1.141	1.060–1.228	1.113	1.046–1.185	1.089	1.039–1.142
Alcohol drinker						
No	1.000	-	1.000	-	1.000	-
Yes	1.034	0.950–1.125	1.002	0.944–1.065	1.140	1.023–1.270
Ethnicity						
Han	1.000	-	1.000	-	1.000	-
Yi	0.898	0.702–1.149	1.095	0.654–1.833	1.413	1.003–1.992
Miao	0.991	0.790–1.243	0.963	0.793–1.170	0.835	0.732–0.953
Mongolia	0.835	0.668–1.042	0.956	0.902–1.013	0.931	0.823–1.053
Tibetan	0.789	0.626–0.995	1.258	1.094–1.446	1.187	1.042–1.351
Korean	1.280	1.046–1.566	1.224	1.089–1.376	1.100	0.960–1.261
Hui	0.985	0.855–1.135	1.056	0.977–1.141	1.121	1.040–1.351
Tujia	0.868	0.722–1.042	1.001	0.807–1.241	1.278	1.148–1.424
Others	1.035	0.885–1.210	0.860	0.676–1.094	1.079	0.899–1.293
Disease family history						
No	1.000	-	1.000	-	1.000	-
Yes	1.200	1.060–1.357	1.212	1.118–1.315	1.308	1.229–1.392
BMI						
Normal	1.000	-	1.000	-	1.000	-
Overweight	0.976	0.919–1.038	0.929	0.880–0.980	0.946	0.900–0.993
Obesity	0.948	0.850–1.058	0.926	0.860–0.997	0.966	0.902–1.033
Seep duration						
≥6 h	1.000	-	1.000	-	1.000	-
<6 h	1.194	1.068–1.335	1.215	1.094–1.350	1.158	0.986–1.359
Sleep quality						
Good	1.000	-	1.000	-	1.000	-
Poor	1.534	1.357–1.733	1.507	1.377–1.650	1.774	1.485–2.120
Stress						
No	1.000	-	1.000	-	1.000	-
Yes	2.346	2.054–2.681	1.284	1.240–1329	1.459	1.378–1.544
Negative life event						
No	1.000	-	1.000	-	1.000	-
Yes	1.277	1.144–1.425	1.104	0.885–1.376	1.197	0.917–1.563
Positive life event						
No	1.000	-	1.000	-	1.000	-
Yes	1.257	1.133–1.394	0.987	0.726–1.342	0.779	0.695–0.874
Hypertension						
No	1.000	-	1.000	-	1.000	-
Yes	0.949	0.887–1.016	1.016	0.977–1.057	0.978	0.932–1.026
Regular exercise						
No	1.000	-	1.000	-	1.000	-
Yes	0.873	0.720–1.060	0.910	0.825–1.004	0.897	0.766–1.049
Diet choice						
Routine	1.000	-	1.000	-	1.000	-
Unhealthy	1.037	0.927–1.160	1.107	1.003–1.221	1.069	0.962–1.187
Meal time						
Regular	1.000	-	1.000	-	1.000	-
Irregular	1.505	1.404–1.612	1.168	1.062–1.284	1.299	1.118–1.509

OR: odds ratio; CI: confidence interval; GEE: generalized estimating equation; Family history: family history of cardiovascular or cerebrovascular diseases. The social adaptability dimension contains two items: feeling tired at work and incompatibility with coworkers. The immunity dimension contains one item: susceptibility to flu or other diseases. The going to hospital dimension contains one item: the feeling of suffering from undiagnosed diseases.
